# Nasal septal perforation 1981–2005: Changes in etiology, gender and size

**DOI:** 10.1186/1472-6815-7-1

**Published:** 2007-03-07

**Authors:** Liv Kari Døsen, Rolf Haye

**Affiliations:** 1 Department of Otolaryngology, Rikshospitalet-Radiumhospitalet HF, University of Oslo, 0027 Oslo, Norway

## Abstract

**Background:**

Septal perforation is an uncommon but very bothersome illness and treatment is difficult particularly with large perforations. We wanted to establish the etiology and size of nasal septal perforations in an attempt to implement preventive measures.

**Methods:**

This is an open, prospective clinical study of patients seen at our hospital from 1981 to 2005. The clinical data of size, gender and etiology have been recorded consecutively.

**Results:**

One hundred and ninety seven patients (100 male, 97 female) were evaluated. Between 1981 and 1995 nasal septal perforation was caused by surgery in 40 of 102 (39.2 %). In the period 1995 to and inclusive of 2005 this percentage decreased as septal resection has been replaced by septo/septorhinoplasty. The latter was the cause for septal perforation in 14.7% in the last period. Nasal steroid and decongestive sprays have emerged as an important cause (28.4 %) during the last ten years particularly in females. In the first period 44 (43.1 %) and in the last 53 (55.7 %) patients were females. There was a noticeable reduction in the number of septal perforations 15 mm or larger in the last period.

**Conclusion:**

Nasal steroid and decongestive sprays are now important causes for septal perforation. Information about this complication should be given with an advice to immediately report increasing and bothersome crusting and bleeding. Warning of the simultaneous use of nasal steroid and decongestive sprays should be addressed particularly to females. All patients with symptoms of septal perforation should promptly be referred to otolaryngologists for treatment.

## Background

The treatment of nasal septal perforation (SP) is symptomatic (local application of ointments), prosthetic, or surgical. Surgery may be curative but the results are not always satisfactory as evidenced by the many different operative procedures that have been proposed [1-7]. Prevention of SP should be an important goal. The purpose of this study was to examine our clinical data to look for possible changes in the pattern of etiology in order to identify causes that may be suitable for preventive measures. We have also registered the size of the SP in order to establish whether the size would indicate that the patients should have been referred sooner.

## Methods

We have recorded the etiology, symptoms and the size of SP from 1981 to and inclusive of 2005. To establish the cause of a SP we have relied on the case history, the time and the description of prior surgery, observations by otolaryngologists and careful consideration of the timing of symptoms in relation to the start of medication. For this study we have not included patients with Wegener's granulomatosis. In some cases we were unable to find any specific cause for or contributing factor to the development for the SP and have therefore labelled them unknown. Measurement of the perforations was done with malleable pins vertically (perpendicular to the nasal floor) and horizontally. In 1995 we did an interim analysis of patients being treated from 1981 to1995 (unpublished).

There is no ethical question involved as the study is non-experimental and only anonymous data have been used.

## Results

We have seen 197 patients with symptomatic SP, 100 males with a mean age of 40.6 and 97 females with a mean age of 41.3. In the first period there were 58 males and 44 females, whereas in the second period there were 42 males and 53 females. The causes for SP from the cases seen from 1981 to 1995 are compared to those observed from 1996 to 2005 (Table 1). Causes compared to gender are listed in Table 2. There was no predilection in brand or delivery system of nasal steroid sprays. In a few patients the SP occurred within three months after the start of the application of a nasal steroid. We have only seen one patient in whom cocaine use was the cause. Septal deviation was seen in 48% of the patients. Nineteen patients in the first period had a SP 15 mm and larger whereas only three in the last period. Twenty nine (30 %) had a SP ten to 14 mm in this period. There was no difference in the size of the SP according to its cause.

**Table 1:** Causes of septal perforation according to time period

Time interval	1981–1995		1996–2005		Total
	No	(%)	No	(%)	No

Septal resection (Killian)	22	(76)	7	(24)	29
Septo/septorhinoplasty	18	(56)	14	(44)	32
Trauma	18	(58)	13	(42)	31
Cautery	9	(47)	10	(53)	19
Nasal picking	6	(50)	6	(50)	12
Nasal steroids	1	(7)	14	(93)	15
α-adr. nasal spray	0	(0)	2	(100)	2
α-adr. + steroid sprays	0	(0)	11	(100)	11
Tamponade	1	(100)	0	(0)	1
Other	4	(67)	2	(33)	6
Unknown	23	(59)	16	(42)	39
Total	102	(52)	95	(48)	197

**Table 2:** Causes of septal perforation according to gender

	Total	Male	Female
Septal resection (Killian)	29	22	7
Septo/septorhinoplasty	32	23	9
Trauma	31	15	16
Cautery	19	10	9
Nasal picking	12	5	7
Nasal steroids	15	5	10
α-adr. nasal spray	2	1	1
α-adr. + steroid sprays	11	0	11
Tamponade	1	1	0
Others	6	1	5
Unknown	39	17	22
Total	197	100	97

## Discussion

Although this study has been ongoing for 24 years the senior surgeon has been responsible for continuously registering the data during the whole study period. Thus these data should be comparable throughout.

The main cause for SP was due to nasal operative procedures in first period particularly among men as seen in other studies [8,9]. These were, however, a less frequent cause in the last period. The submucosal nasal septum resection technique (Killian) has been reported to cause SP in 17% compared to 1.3% after septo/septorhinoplasty [10]. As the former procedure has not been performed in Norway after 1990, this explains the reduction of surgery as a cause. As septo/septorhinoplasty was the cause in 14.7 % of the cases in the later part of the study, we think it is still important to emphasize the need for meticulous care during surgery.

The higher frequency of males in the group caused by surgery is probably due to a higher frequency of men undergoing nasal surgery. In 1985 nasal sprays were reported to cause SP [11]. Although listed as a possible cause in a review by Kridel [1], this etiology has not been recorded in surgical studies neither before nor after 1995 [2,3,7-9,12]. Perhaps direct questioning may uncover such cause. The increase in nasal sprays as a cause seen in the last period of our study may reflect the more widespread use of nasal steroids. Of particular interest is the finding that they are a more important cause in females than in males as also reported by Cervin and Andersson [13]. This was particularly evident when nasal steroid and decongestive sprays were used simultaneously. As SP was seen in patients using only inhaled steroid powder, we do not believe that the spraying as such is the cause. The reason for the difference in gender is not apparent. Septal deviations were less frequent in females and cannot account for this difference. In this study there was a switch in the proportion of males and females with a majority of females in the last period. This tendency has not been observed in other studies [2,8,9]. The reason for this is not evident, but may be associated with the changing pattern in etiology.

We believe that it should be appropriate to inform patients of SP as a complication of nasal steroid sprays and advice them to report the occurrence of bothersome or increasing crusting and/or bleeding as soon as possible. These cases should promptly be referred to otolaryngologists. Early intervention may prevent the occurrence of SP. Female patients should be warned of the danger of using them simultaneously with decongestive nasal sprays for a longer period.

Presently we see few of the large SPs, so many are now amenable to surgical treatment. We would, however, like to be able to intervene earlier. Better information to general practitioners might ensure this.

## Conclusion

Nasal septal surgery used to be the main cause for SP, particularly in men. Better surgical techniques seem to have reduced but not eliminated this risk. Nasal steroid and decongestive sprays are now important causes for SP. Information about this complication should be given with an advice to immediately report increasing and bothersome crusting and bleeding. Warning of the simultaneous use of nasal steroid and decongestive sprays should be addressed particularly to females. All patients with symptoms of SP should promptly be referred to otolaryngologists for treatment.

## Abbreviations

SP Septal perforation

## Competing interests

The author(s) declare that they have no competing interests.

## Authors' contributions

LKD has compiled the data and has the main responsibility for the manuscript. RH has registered the clinical data continuously and has participated in the writing of the manuscript.

Both authors have read and approved the final manuscript.

## Pre-publication history

The pre-publication history for this paper can be accessed here:


